# A new linear combination method of haplogroup distribution central vectors to model population admixtures

**DOI:** 10.1007/s00438-022-01888-0

**Published:** 2022-04-11

**Authors:** Tibor Török, Kitti Maár, István Gergely Varga, Zoltán Juhász

**Affiliations:** 1 Department of Genetics, University of Szeged, Szeged, Hungary; 2 Department of Archaeogenetics, Institute of Hungarian Research, Budapest, Hungary; 3 Department of Pediatrics and Pediatric Health Center, University of Szeged, Szeged, Hungary; 4 Institute of Technical Physics and Materials Science, Centre for Energy Research, Budapest, Hungary

**Keywords:** Archaeogenetics, Haplogroups, Artificial intelligence, Self learning

## Abstract

We introduce a novel population genetic approach suitable to model the origin and relationships of populations, using new computation methods analyzing Hg frequency distributions. Hgs were selected into groups which show correlated frequencies in subsets of populations, based on the assumption that correlations were established in ancient separation, migration and admixture processes. Populations are defined with this universal Hg database, then using unsupervised artificial intelligence, central vectors (CVs) are determined from local condensations of the Hg-distribution vectors in the multidimensional point system. Populations are clustered according to their proximity to CVs. We show that CVs can be regarded as approximations of ancient populations and real populations can be modeled as weighted linear combinations of the CVs using a new linear combination algorithm based on a gradient search for the weights. The efficacy of the method is demonstrated by comparing Copper Age populations of the Carpathian Basin to Middle Age ones and modern Hungarians. Our analysis reveals significant population continuity since the Middle Ages, and the presence of a substrate component since the Copper Age.

**Supplementary Information:**

The online version contains supplementary material available at 10.1007/s00438-022-01888-0.

## Introduction

Nowadays, we witness a rapid accumulation of modern and ancient human DNA data, which combined with the development of sequence analysis methods opens new perspectives in studies of human prehistory (Skoglund and Mathieson 2018). Although full genomes undoubtedly provide the greatest information for studying relationships between individuals and populations, Y-chromosomal and mitochondrial data remain essential due to their uniparental inheritance and lack of recombination (Kivisild 2015). Ancient and modern human populations can be easily characterized by their Hg frequency distribution vectors, and the computational analysis of these distributions may reveal hidden relationships due to early admixture, separation and migration processes. The incentive of our work was to elaborate such computational methods.

Comparison of the maternally inherited mitochondrial DNA distribution of populations is widely used in population genetic analyses. The standard approach is fixation index (F_ST_) statistics implemented in Arlequin (Excoffier and Lischer 2010) that measures the genetic differentiation between populations calculated from nucleotide diversity and also incorporating evolutionary distance between haplotypes (Excoffier et al. 1992). Pairwise Fst distance matrices or haplogroup (Hg) frequency matrices can be visualized on MDS or PCA plots, where similar populations are expected to cluster close to each other. As distance matrices of vectors can easily be generated, all distance-based methods are suitable for clustering multidimensional vector data.

There are alternative ways to cluster multidimensional vector data, like k-means (Hartigan and Wong 1979), which is based on an essentially different principle, searching directly for central vectors of the local condensations of the multidimensional point systems defined by the vector data. Thus, the primary product of vector-based methods is a set of central vectors (CVs) of the local condensations, and the clusters are determined after, as sets of data vectors being in the vicinity of the nearest CV. The main advantage of vector-based methods is the CV set, since CVs represent the common features characterizing all cluster members by their mean. CVs can be calculated with the vector-based “Self-Organizing Cloud” algorithm successfully applied in previous studies (Juhász et al. 2015).

As CVs themselves are also vectors in the vector space of the basal Hg set, a given CV can be interpreted as a hypothetical “ancestral population” with descendants constructing its own cluster. Indeed, we show here that CVs in our vector space model really can be well interpreted as ancient source populations of prehistorical migrations. However, the Hg-distribution vectors in our database construct a rather fuzzy point system in the space of the basal Hg set; therefore, an interpretation of a population as the descendant of the nearest CV may be an extreme simplification. Instead, the mathematical model describing the data vectors as linear combinations of all of the CVs with different weights is a more adequate representation. In this model, populations are interpreted as admixtures of the hypothetical ancestor populations represented by the CVs. We show in this paper a new gradient search algorithm determining the weights constructing the optimal models of the data vectors as linear combinations of the CVs.

To study the relationships of ancient and modern human populations, we generated a new mitogenome database from published data, then defined a reduced set of Hgs playing the most conspicuous roles in early migration and admixture processes. The selection of this set was based on the hypothesis that the footprints of the most important early migration processes are found in associations of Hgs whose frequencies show correlated variations in several groups of populations. The “iterative rank correlation” method has been based on this hypothesis and successfully applied in several previous works (Juhász et al. 2018[7]. Therefore, we assume that the set of Hs showing correlated propagation with other Hgs provides a common basis for determining Hg frequency distribution vectors in a common vector space.

## Methods

### Construction of a common Hg basis for universal description of populations in the database

Our database contains the mitogenome Hgs of 15,919 individuals belonging to 62 modern and 115 ancient populations (Supplementary Table 1). The Hgs appearing in the database are classified into 4159 sub-Hgs which are labeled by 1–17 characters. Thus, each population can be described by distribution vectors containing the frequencies of their own Hgs, but the sizes and Hg contents of Hg sets characterizing different populations are necessarily different. To make a mathematically sound basement to reveal genetic relations between populations, we selected a common subset of the 4159 Hgs which occur in multiple populations with a significant frequency.

As the label system of the Hgs mirrors the tree structure of the subclades and deep subclades with long labels seldom occur in many populations, it is obvious to define a maximal depth of the Hgs specificators to be added to the common set. Defining this maximal number as 3 (see Results), we obtained 654 Hgs with depth of one to three characters (e.g., H, H1, H1a, H13a, H11ab). By eliminating the Hgs with very low prevalence, (frequency of 0.0005 within the total database), this value was further reduced to 224. Considering the resulting set of Hgs as a common basis for the whole dataset, an Hg distribution can be generated by classifying each member of a given population into one of its best matching group present in the common 224 Hg set. This way, we can preserve the information contained in the phylogenetically deeply classified Hgs with longer sizes of labels. For example, let the common Hg set be A, B, A1, B2a, and the Hgs appearing in a population A2a1, A1b, B2a3, B. In this case, Hg A2a1 is ordered to Hg A, Hg A1b to Hg A1, Hg B2a3 to Hg B2a, and, obviously, Hg B to Hg B.

The next step in the construction of an optimal common basis of Hgs is based on the assumption that the frequencies of Hgs jointly taking part in the most important early migration processes show correlated propagation in ancient and modern populations. The relevance of this assumption has been discussed and validated in earlier publications [7]. The correlated propagation of Hgs can be indicated using a rank correlation analysis [8] as follows: considering the 224 Hgs as a common basis, we can determine the Hg distributions of our 172 populations one by one. Being in possession of these 224-dimensional (224D) Hg distributions, we can determine the rank list of, e.g., Hg A by ordering the 172 populations according to their frequencies of Hg A. We can construct such rank lists for all of the 224 Hgs and calculate the rank correlations for each Hg pair. Selecting the Hgs having at least one pair with a rank correlation value exceeding 0.8, we obtain an Hg collection reduced to the most important jointly spreading Hgs, suitable for studying migration processes. Obviously, correlations over 0.8 cannot be expected in the whole set of the 172 Eurasian populations because of the dynamic population processes of the past 10,000 years. We used our “iterative rank correlation algorithm” to accomplish a systematic search for subsets of populations in which high correlation can be detected between Hg pairs, and we accepted correlations over 0.8 if these were detected in at least ten populations. In this way, we finally obtained a 74-element basis of correlating Hgs with maximum depths of three phylogenetic labellings (Table 1). Correlation values exceeding 0.8 and/or critical population number above 10 resulted in a radical reduction of the number of suitable Hgs. For example, increasing the critical number of populations to 15, the number of suitable Hgs decreased by ~ 40%.

**Table 1:** The 74 Hgs linking populations and constituting the common basis for the 172 Hg-distribution vectors in our study

1 character	2 characters	3 characters
		C4a, C4b
		D4b, D4e, D4j
		D5a
		F1b
		G2a
H	H1	H1a, H1b, H1c, H1e
		H2a
	H3	H3h
		H4a
	H5	H5a, H5b
		H6a
	H7	H7b
		H11a
		H13a
HV		HV0a, HV1a, HV2a
		I1a
	I2	
		I4a, I5a
		J1b, J1c, J1d
		J2a, J2b
		K1a, K1b, K1c
		K2a
		L2a
		M1a
		N1a, N1b, N9a
		R0a, R1a
		T1a
	T2	T2a, T2b, T2c, T2e, T2f
		U1a
		U2e
		U3a, U3b
		U4a, U4b, U4c, U4d
		U5a, U5b
		U8a, U8b
V		
		X2b, X2c, X2e
		W3a

### Determination of the set of local condensation centres of the 74-dimensional Hg-distribution vectors representing our 172 populations

The 172, 74-dimensional Hg-distribution vectors representing 172 populations create a point distribution in a common 74-dimensional vector space, where groups of similar Hg-distribution vectors—representing genetically similar populations—form condensations. Our next purpose was to determine the central vectors of all of these condensations. Our tool for this purpose is the unsupervised learning system “Self-Organizing Cloud” (SOC) algorithm described in several past publications [6]. In our case, the training (input) vectors of the SOC are the 172 Hg distributions, while the learning (output) vectors are the condensation centres. The SOC algorithm also determines a sufficient number of the learning vectors *N,* for describing the clusters of the given vector system with an appropriate significance. The SOC algorithm automatically increases the number of the learning vectors until the significance of the clustering exceeds a predefined critical value. In our case, this was formulated in the condition that the average distance of a cluster centre from the cluster members (the “radius” of the cloud constructed by the cluster) should be less than 1/3 of the distance from the nearest neighbouring cluster centre. Thus, the set of the resulting local condensation central vectors (CVs) provides an optimal and adequate model for the basic structure of the 172 Hg distributions. Being in possession of these CVs, the original Hg distribution can be arranged into independent clusters by ordering each Hg distribution to its nearest CV.

### Modeling the Hg distributions as weighted admixtures of local condensation central vectors

Although the above approach attributes each Hg distribution to one cluster unambiguously, the fuzzy structure of the point system makes it possible to relate an Hg distribution to more CVs simultaneously, with different weights depending on the distances of the CVs from the given Hg distribution. The mathematical problem can be formulated as follows. We approximate the given D-dimensional vector $\underline{h}$ as a weighted sum of the set of *N* given D-dimensional vectors $\underline{v}_{1} ...\underline{v}_{N}$:

1 $$\underline{h} = a_{1} \underline{v}_{1} + a_{2} \underline{v}_{2} + \ldots + a_{N} \underline{v}_{N} + \underline{\varepsilon } ,$$

where $\underline{h}$ is the Hg-distribution vector to be approximated by the CVs $\underline{v}_{1} ...\underline{v}_{N}$, *N* = *74* is the number of the CVs and $\underline{\varepsilon }$ is the error vector of the approximation. Our aim is to find the optimal set of the weights $a_{1} ...a_{N}$, minimizing the power of the error vector $\underline{\varepsilon }$ (the squared sum of the *D* error components):

2 $$H = \varepsilon_{1}^{2} + \varepsilon_{2}^{2} + \ldots + \varepsilon_{D}^{2} = \sum\limits_{k = 1}^{D} {\varepsilon_{k}^{2} = \min } ,$$

where *H* is the power of the error to be minimized and $\varepsilon_{1} ...\varepsilon_{D}$ are the coordinates of the D-dimensional error vector $\underline{\varepsilon }$. Note that the CVs $\underline{v}_{1} ...\underline{v}_{N}$ are usually not orthogonal; therefore, the weights $a_{1} ...a_{N}$ cannot be interpreted as independent coordinates.

The accuracy of an estimation is indicated by the error power *H* normalized by the total power of the vector to be estimated $\underline{h}$:

3 $$J = H/\sum\limits_{k = 1}^{D} {h_{k}^{2} } ,$$

where the accuracy *J* is 0 when the estimation is totally perfect and 1 if the estimation is unsuccessful, i.e. all the weights $a_{1} ...a_{N}$ equal 0.

The partial derivatives of the error power can be analytically formulated as

4 $$\frac{\partial H}{{\partial a_{m} }} = \sum\limits_{k = 1}^{D} {2\varepsilon_{k} \frac{{\partial \varepsilon_{k} }}{{\partial a_{m} }} = 2\sum\limits_{k = 1}^{D} {\varepsilon_{k} ( - v_{m,k} )} } .$$

The numerical solution and the algorithm can be based on the gradient search principle, as detailed in Supplementary text1.

Thus, substituting a real Hg-distribution vector into $\underline{h}$, our algorithm determines the optimal weights $a_{1} ...a_{N}$ for interpreting the corresponding population as an admixture of *N* CVs or “hypothetical ancestral populations” representing *N* clusters of inherently similar real populations. Assuming that the *N* clusters characterized by their central vectors can be well explained from an archaeogenetic point of view, the interpretation of real populations as their weighted admixtures may provide instructive consequences.

## Results

### Generation of Hg-distribution vectors of the populations

Using the method described in Chapter 3.1, we have identified 74 Hgs, constituting the final 74 -element common basis for our analysis. The Hg content of this basis is listed in Table 1 and Supplementary Table 2 and their frequencies in the 172 populations are given in Supplementary Table 3.

Obviously, members of our mitogenome database not belonging to any of the Hgs or their subclades in Table 1 are eliminated from our analysis. (For example, Hg B and its subclades are totally absent in our set.) Moreover, the specific Hgs of the three populations (Com, Gil, Pal_As) could not be assigned to any of the 74 basal Hgs; therefore, we could not classify them (Com, Gil, Pal_As). One of the reasons for this is certainly the incompleteness of the available mitogenome dataset, as Hgs missing from Table 1 were described from few populations. In spite of our effort to collect all available mitogenome data, the eliminated Hgs are not yet informative enough to identify the population relations and main migration processes we wish to study. Independently of the incomplete representation, any definition of a universal Hg basis as a subset of the total Hg set causes more or less distortion of the original distributions. We will show in the next chapter that our correlation-based method provides a sub-optimal definition providing a good correlation with Fst distances in general.

The Hg distributions of the 172 populations determined by this 74-element Hg set can be considered as 172, 74-dimensional vectors in a 74-dimensional orthogonal vector space, where the coordinate axes correspond to the 74 Hgs, and the coordinates of the Hg-distribution vectors are defined as the frequencies of the 74 Hgs in the corresponding population.

### Performance test

To test the suitability of our approach, we compared genetic distances of real ancient populations, measured as unweighted Euclidean distances of our 74-dimensional Hg-distribution vectors to the corresponding Fst distances. For this end, we selected 74 ancient populations from our database, for which both Euclidean and Fst distances could be determined: the test populations are listed in Supplementary Table 4.

Next, we calculated the Euclidean distances of the mtDNA Hg distributions as follows:

5 $$d_{m,n}^{{}} = (\sum\limits_{k = 1}^{D} ( h_{m,k} - h_{n,k} )^{2} )^{1/2} ,$$

where *d*_*m,n*_ is the distance of populations denoted by their serial numbers *m* and *n*, *D* = *74* is the size of the set of the Hg basis, and $h_{m,k}$ and $h_{n,k}$ are the *k*th coordinates of the *m*th and *n*th Hg distributions.

We represented the distances of the above 74 test populations, calculated from Eq. 5 in a symmetric distance matrix of size of 74*74**.**

Another distance matrix—the “reference matrix” of the test—was constructed from the Fst distance data of the same 74 populations. Both distance matrices are given in Supplementary Table 5.

We obtained 0.68 correlation value of the two matrices, while the Mantel test [9] showed that a random permutation of the rows of a distance matrix resulted in an increase of correlation with a probability below 0.0001. These results verify that our Hg distribution-based method may reproduce the results of the standard Fst distance calculation with a good significance; thus, our abridged, jointly spreading 74-element common Hg basis is a suitable representation of the original population. Consequently, the comparison of Hg distribution-based and Fst-based distance data of the test populations has validated our common 74-dimensional Hg basis described in Table 1.

Although the selection of the common Hg basis has been successfully validated by the performance test, its compilation poses several questions. We restricted the common Hg basis to Hgs with label depths of 1–3, even though using deeper phylogenetic subclades with long labels should be clearly favorable in any analysis. Truncating longer Hgs inevitably leads to information loss, which could be reduced by applying higher maximal label lengths in the common Hg basis. On the other hand, using too deep phylogenetic subclades may obscure population relations, as deep Hg subclades are seldom present in numerous populations. This problem is especially obvious when comparing Hg distributions of ancient and modern populations, as most young subclades (with long Hg labels) were not yet present in the ancient populations. Thus, the optimal Hg phylogenetic depth of the common Hg basis needs to be determined.

Another related problem we should consider is how to handle phylogenetically deeply identified Hgs, which are longer than the predefined common Hg set. In our previous analysis, we added these to their nearest Hg present in the common set—which we term “cumulating method”—though in many cases phylogenetic subclades may show independent population genetic dynamics from their parental clades.

Another alternative possibility would be to completely eliminate all subclades over the predefined phylogenetic depth, which we call “cutting method”. While applying the “cutting method” the information contained in the Hgs of the neglected individuals is totally lost, it is at least partly utilized in the former “cumulating method”. It follows that next to determining the optimal phylogenetic depth, we also need to consider the optimal way to handle deeper subclades with longer labels than the selected optimum length for our analysis.

We designed an experiment in which both of the above problems could be tested. To find the optimal Hg length, we reassembled the common Hg set with different predefined Hg label depths and repeated the performance test with each, both with the “cumulating” and “cutting” methods, then we determined the correlations of the corresponding distance matrices with the Fst distance matrix as follows:

1. Most frequent Hgs (all Hgs with database frequency above 0.0005) having maximal depth of 3, the frequency calculated with the cumulative method:
  *N* = 224. Correlation with Fst distance matrix: 0.63, p(Mantel) <  = 0.00001).
2. Correlating Hgs (selected as described in Methods 3.1), having maximal depth of 3, the frequency calculated with the cumulative method (this has been shown in the performance test):
  *N* = 74. Correlation with Fst distance matrix: 0.68, p(Mantel) <  = 0.00001).
3. Most frequent Hgs having maximal depth of 3, frequency calculated with the cutting method:
  *N* = 236. Correlation with Fst distance matrix: 0.28, p(Mantel) <  = 0.00001).
4. Correlating Hgs having maximal depth of 3, frequency calculated with the cutting method:
  *N* = 16. Correlation analysis was eliminated due to the low size of the Hg set.
5. Most frequent Hgs having maximal depth of 4, frequency calculated with the cumulativeg method:
  *N* = 223. Correlation with Fst distance matrix: 0.49, p(Mantel) <  = 0.00001).
6. Correlating Hgs having maximal depth of 4, frequency calculated with the cumulative method:

*N* = 80. Correlation with Fst distance matrix: 0.68, *p* p(Mantel) <  = 0.00001).

The low correlations and set size in Experiments 3 and 4 reveal that the cutting method results in lower correlation with the Fst data than any of the cumulative methods, thus we did not test the cutting method in other combinations.

We endeavored to keep the number of the “most frequent Hgs” defined by frequency threshold 0.0005 comparable in experiments 1, 3 and 5. Experiments 1 and 5 reveal that the maximal depth of 3 is preferable than 4 when using the cumulative method.

As Experiments 2 and 6 led to the highest correlations with Fst, thus the selection of correlating Hgs subset proved to be the most effective approach with maximal depths of 3 and 4. Increasing the depth to 5 or above, we obtained a radical loss of correlations.

It is remarkable that the correlating 74 and 80 Hgs in experiments 2 and 6 comprise subsets of the “most frequent Hgs” in experiments 1 and 5, respectively, nevertheless gave better results.

Thus, we can conclude that the best choice for our study is Experiment 2, the reduction of the Hg set to correlating Hgs and applying the cumulative method with maximal Hg depth of 3.

### Clustering of the Hg distributions

As we have mentioned in Methods 3.2, the SOC algorithm automatically increases the number of learning vectors until the significance of the clustering exceeds a predefined critical value. To satisfy this requirement for our dataset, the algorithm increased the number of CVs up to 35. Using the Student’s t test, we found that the probability that the nearest CV does not belong to the studied cluster is more than 0.99 for all cases. The correlation between the inherence matrix and the distance matrix of the 172 populations is 0.4. (As the element (i,j) of the inherence matrix is 1 if the ith and jth elements of the data set belong to the same cluster and 0 otherwise, negative correlations with the distances indicate good correspondence here.) The populations constructing the 35 clusters are listed in Supplementary Table 6. The list shows that the SOC attributed “own” clusters to certain populations having very specific Hg distributions.

#### Demonstrating the biological relevance of CVs

**Fig. 1:**
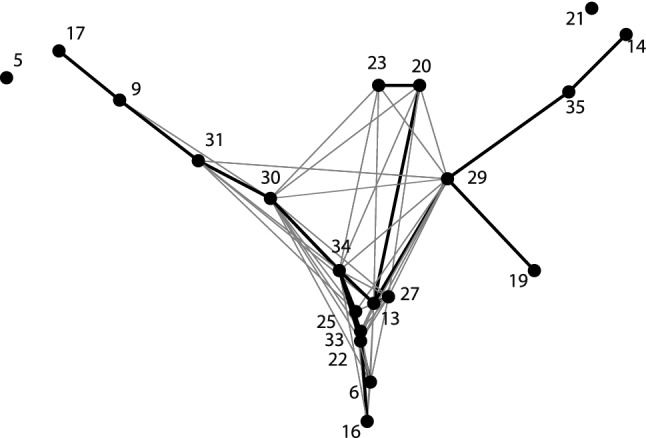
MDS map mirroring the relationships of the most related 20 CVs from the total 35-element CV set. Outlier CVs not related to the main cluster are not represented. Thin edges connect CVs with Euclidean distances below 35% of the maximal value, while the thick edges indicate the minimal spanning tree of the system, connecting the 20 populations with minimal sum of distances

We represented the relationships of the 35 CVs on the MDS plot, which revealed that 20 CVs form a genetic network connected by significantly close genetic distances, while the remaining 15 CVs are outliers, which do not seem to be part of this network. The main relationships of the 20 CVs are represented by the MDS map in Fig. 1, with the exclusion of 15 outliers.

The tree-like structure of Fig. 1 has an inner network with three main protruding branches. Next, we will show that CVs have well interpretable genetic relations, as the branches correspond to well-defined ancient populations, while the inner network represents populations with various admixture events from the branches.

##### European hunter–gatherers, upper left branch

**Fig. 2:**
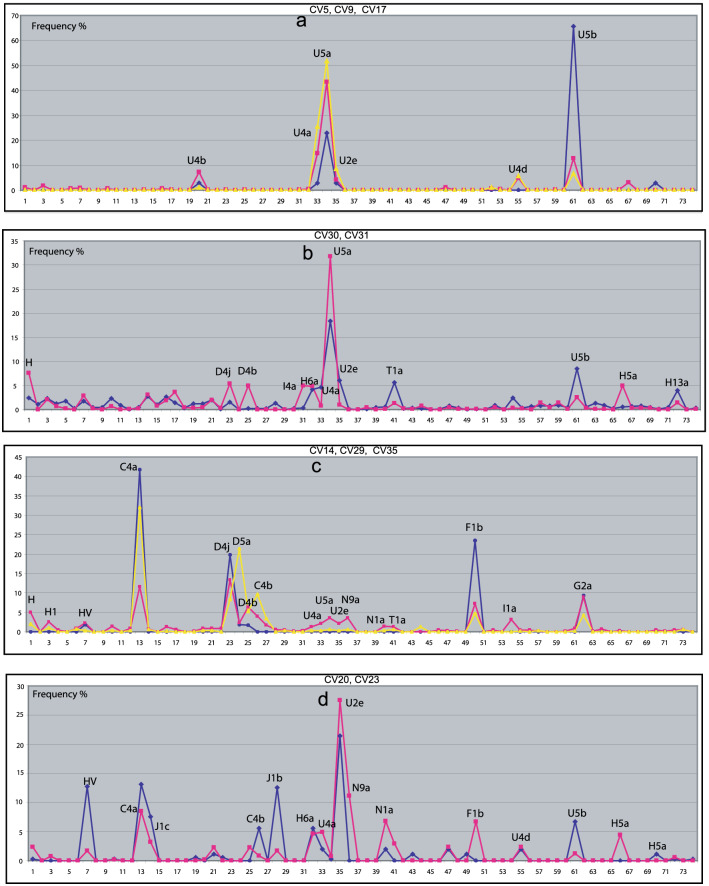
Hg distributions of selected CVs. **a** CV5 (blue), CV9 (red), CV17 (yellow); **b** CV30 (blue), CV31 (red); **c** CV14 (blue), CV29 (red), CV35 (yellow); **d** CV20 (bue), CV23 (red). The horizontal axis shows Hg serial numbers, according to Supplementary Table 2. The vertical axis shows Hg frequencies. Most frequent Hgs are labelled at the peaks (colour figure online)

The left upper branch ion Fig. 1 includes CVs 5, 17, and 9, and their Hg distributions are shown in Fig. 2a. All of the populations in these clusters are European Mesolithic hunter–gatherers (Supplementary Table 6) belonging to Hgs U2e, U4a, U4b, U4d, U5a and U5b, with the dominance of U5a and U5b. The closest Hg distributions to CVs 5 and 9 belong to Western hunter–gatherers (WHG) and Ukrainian Neolithic (Ukr_N).

CV 30 and 31 connect the hunter–gatherer branch to the inner network suggesting that these CVs may contain populations with additional elements on an HG layer and indeed these CVs include European and steppe Bronze Age–Iron Age populations besides modern Eastern Europeans (Supplementary Table 6) in which this admixture has been documented [10–12]. The similarity between Figs. 2a, b is conspicuous, four of the five Hgs (U5a, U2e, U5b, U4a) are common, but in CV30 (Fig. 2b blue line) the hunter–gatherer substrate is completed by H6a, T1a and H13a. We will show below that Hgs T1a and H13a play the most important role in Caucasian and Near Eastern populations, so CV30 may mirror a contribution of ancient Near Eastern, and/or Caucasian populations to a hunter–gatherer substrate as has been shown before [13]. As the closest ancient populations to CV30 are Baltic Iron Age (Balt_IA) and Mierzanowice Bronze Age (Mrz_BA), CV30 may be attributed to an ancient North-Eastern European Bronze Age population.

CV31 is also dominated by the hunter–gatherer U5a, and U5b Hgs (Fig. 2b red line), but in this group further Western Eurasian and Asian components are represented by Hgs H, H5a, H6a, I4a, as well as D4j and D4b. As both Yamnaya–Afanasievo (YamAf) and Karasuk (Kar) belong to CV31, this may refer to the contribution of the East European Yamnaya to the South Siberian Karasuk population [14].

##### Asian populations, upper right branch

The upper right branch on Fig. 1 includes CVs 21, 14, 19, 29 and 35, all of which represent Asian populations (Supplementary Table 6). CV21 corresponds to modern Siberians: even Nganasan and Yukaghir. CV19 contains modern East-Asians: Japanese, Chinese and Tuvinian, CV14 Bronze Age Siberians and CV35 modern and ancient East-Inner Asian populations, while CV29 links these groups to the rest of the tree. We analyze here CVs 29 to show that it represents Hgs with European links and CV35, as an example of the Asian groups with no Western Eurasian contacts.

Hg distribution of CVs 29 and 35 are shown in Fig. 2c.

The hunter–gatherer component indicated by U2e, U4a and U5a is also detectable in CV29 (Fig. 2c red line) together with low prevalence European Hgs H, H1, HV, T1a and I1a. However, the dominant components are here Asian Hgs C4a, C4b, D4b, D4j, F1b and G2a. This refers to a stable Asian substrate with European contribution and, accordingly, CV29 also contains Scytho-Siberians, Saka, TienShan Huns and Xiongnus, all of which are known to be Steppe_MLBA–Siberian admixed populations with considerable European elements [11, 15, 16]. That is why CV29 links Siberian and Western Eurasian populations. Population with the closest Hg distribution to CV29 is Baikal Neolithic (Baik_N), indicating a substantial contribution of Baik_N to the above populations in agreement with the cited genomic results.

CV35 (Fig. 2b yellow line) represents another Asian group with predominance of Hgs C4a, C4b, D4b, D4j, D5a, F1b, and G2a, without Western Eurasian component. The closest populations to CV35 are Inner-East Asia Neolithic (IEA_N,) including samples from China, Mongolia and Far Eastern Russia (DevilsCave) as well as modern Yakuts and Tibetians. It has been shown that DevilsCave (IEA_N) genomes contributed significantly to Baikal Neolithic (Shamanka, Lokomotiv, Ust'-Ida [17], which may provide the direct link between CV35 and CV29.

##### Central Asian–South Siberian groups, upper middle branch

CVs 20 and 23 are located in the intermediate area between the two upper branches in Fig. 1, and the edges link these groups both to European (CV30) and Asian (CV29) nodes, which refer to an admixture of Western and Eastern Eurasian components in these Hg distributions. CVs 20 and 23 contain Central Asian–South Siberian Bronze–Iron Age–Medieval populations—Kazakhstan Kangju, Iron Age-Medieval Nomads, Wusun, Sintashta, Tagar, Mongolian Uyuk (Supplementary Table 6), which have been shown to contain these east–west admixtures [11, 16]. We analyze here the Hg distribution of CV23 in detail.

The closest populations to CV23 are the Bronze Sintashta (Steppe_MLBA) and Iron Age Tagar (Scytho-Siberian) populations. The Hgs constructing CV23 (Fig. 2d red line) can be divided into four groups as follows:

1. U2e, U4a, U4d, and U5b which were also found in CVs 5, 9, 17 and 30, mainly attributed to Mesolithic–Neolithic hunter–gatherers.
2. C4a, C4b and F1b were also found in CVs 29, 35 with the closest populations Baik_N and IEA_N, attributed mainly to the South Siberian substrate population.
3. HV, J1c, and N1a which were also found in CVs 13, 16, 25, 33 and 34 will be attributed mainly to Neolithic farmer, as well as Caucasian-Near Eastern populations below.
4. H6a was also found in CV31 Poland Bronze Age (BellB_Pol, Str_BA) dominated by Western Eurasian Hgs arising from Central and Eastern Europe, as well as from the Near East and the Caucasus.

These data altogether indicate that populations belonging to CV23 could be derived from admixtures of the listed components. European farmer, Caucasus, and Eastern hunter–gatherer components of Sintashta genomes have been documented [14], while Scytho-Siberian Tagar genomes were also shown to be admixtures from major Sintashta MLBA and minor Siberian elements [11].

##### Anatolian–European Neolithic, lower branch

CV16 represents the lower extreme of the tree, containing Anatolian and European Neolithic populations (Supplement), while CV22 and CV33 are their closest related groups containing ancient and modern Southern European as well as European Bronze Age groups.

**Fig. 3:**
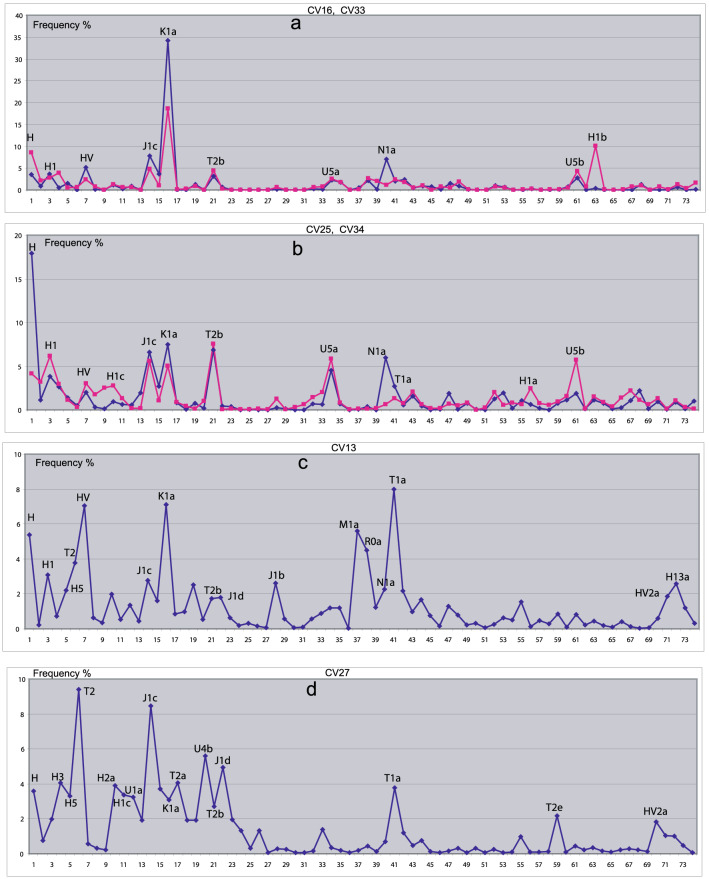
Hg distributions of **a** CVs 16 (blue), CV33 (red); **b** CV25 (blue), CV34 (red); **c** CV13 (blue); and **d** CV27 (blue). Horizontal axis: Hg serial numbers, according to OR Hg-list-74.xls. Vertical axis: Hg frequency. Most frequent Hgs are labeled near the peaks representing their frequencies (colour figure online)

We begin the interpretation with CVs 16 and 33 (Fig. 3a). The Hg set H, H1, HV, J1c, K1a and T2b is totally common in both CVs, while N1a and H1b are specific for CV16 and CV33, respectively. Hgs U5a and U5b also appear simultaneously in both CVs, but their low frequencies may refer merely to a weak influence of Mesolithic–Neolithic hunter–gatherers to the populations represented by CVs 16 and 33. The closest Hg distributions to CV16 belong to Anatolia Chalcolithic (An_CA), Anatolia Neolithic (AN_N), Near-East Neolithic (NE_N) and Great Bitain Neolithic (GB_N), so CV16 can be attributed to European Neolithic farmer populations. The nearest populations to CV33 are Iberian Bronze Age (Ibe_BA) and Hungarian Bronze Age (HU_BA), so CV33 may be considered as the Bronze Age derivative of CV16, modified by genetic drift, bottleneck effect or admixture. Note that Hgs HV, J1c and N1a were also identified in CV23 above, as contributions of a Neolithic farmer gene flow to South Siberia.

The Hg distributions of CVs 25 and 34 (Fig. 3b) display obvious similarities to those of CV16 and CV33. For example, the Hg set of H, H1, HV, J1c, K1a and T2b identified above as common components of CVs 16 and 33 are also present with dominant frequencies in CVs 25 and 34. The main difference between Figs. 3a and 3b are the high frequencies of Hgs U5a and U5b in CVs 25 and 34. This can be interpreted as a balanced admixture of a Mesolithic hunter–gatherer and a Neolithic farmer population. Most similar populations to CV25 are European farmers (EU_LBK), Hungarian Chalcolithic (HU_CA), and Near-East Chalcolithic (NE_CA), whereas modern populations are totally missing from its cluster. At the same time, the closest ancient populations to CV34 are Hungarian Bronze Age Bell-Baker (BellB_HU), Bulgarian Bronze Age (Thr_BA) and Poland Iron Age, (Pol_IA), as well as Baltic Medieval (Balt_Med), Baltic post-Medieval (Balt_PM), and European Medieval (Eu_Med). Thus, CVs 25 and 34 can be identified as earlier and later versions of a population originating from an admixture of a Mesolithic–Neolithic hunter–gatherer and a Neolithic farmer population corresponding to previous data [18]. In addition, very similar modern populations to CV34 are also found in Western and Northern Europe (Ire, Nor, Dan), whereas non-European populations are missing.

##### Caucasus–Near East, lower middle part of the tree

Figure 1 indicates close Euclidean distance of CV13 from CV34 and, indeed, the high prevalence of the Hg set of H, H1, HV, J1c, K1a and T2b links these populations. At the same time Hgs T2, J1b, J1d, M1a, R0a, T1a, HV2a, and H13a provide the particularity of CV13. Most similar ancient populations to CV13 are Near East–Caucasus Neolithic–Chalcolithic–Bronze Age populations (NE_N, NE_CA, NE_BA, Ar_CBA, Ar_IA) clearly showing the geographic roots of CV13. In addition, very close modern populations, modern Iranian (Ira) and Pakistan–Afghanistan (Paf) refer to the same area. Note also that Hgs T1a and H13a were mentioned as Near Eastern–Caucasian contributions to the North-East European CV30-dominated by hunter–gatherer Hgs.

### Demonstrating the efficacy of central vector modeled admixture

Let us assume that the CVs determined by the SOC algorithm represent Hg distributions of 35 hypothetical ancient populations and any of the 172 populations in our study can be modeled as admixtures of these. Based on this assumption, the Hg distribution of an actual population can be modeled as the linear combination of the 35 CVs. The weights of the 35 CVs (hypothetical ancient populations) in the given real population are determined by the admixture algorithm as the coordinates $a_{1} ...a_{N}$ in Eq. 1 (Supplementary Table 7).

Being in possession of the weights determined for a given population, its “CV-modeled” Hg distribution also can be calculated using Eq. 1. By comparing the “CV-modeled” Hg distribution to the real one, we can test the accuracy of the admixture model.

To demonstrate the efficacy of the method we chose to analyze historical populations of the Carpathian Basin as examples.

**Fig. 4:**
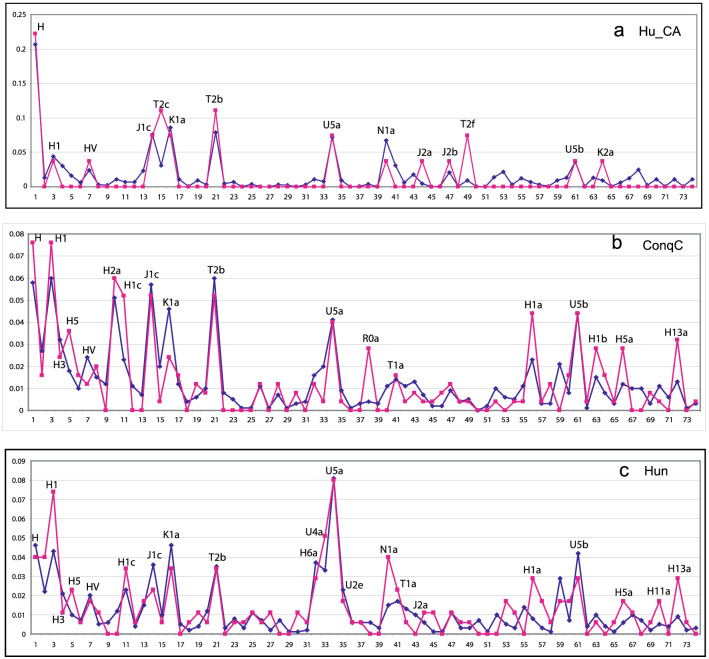
Hg distributions of **a**: Hungarian Copper Age (Hu_CA), **b**: Conqueror commoner (ninth to eleventh century ConqC) and **c**: modern Hungarian (Hun) populations. Red lines indicate original Hg distributions, while blue lines show CV modeled distributions (colour figure online)

We show three examples of the original and the modeled Hg distributions on Fig. 4, for Hungarian Copper Age (Hu_CA), ninth to eleventh century Conqueror commoner (ConqC) and modern Hungarian (Hun) populations from our dataset. Figure 4 illustrates that the original frequencies of the most important Hgs are modeled with an appropriate accuracy by the linear combinations of central vectors. The normalized error of the estimation calculated using Eq. 3 were 0.455, 0.46 and 0.512, respectively, while the average error was 0.515. These values indicate medium or better than medium quality models. For instance, the coincidences of the peaks of the sample and model distributions of Hgs H, H1, HV, J1c, K1a, T2b, U5a, U5b on Figs. 4a–c designate that the performance of the modeling was optimal. The deviances may be explained by the possible lack of certain important ancient populations in the database or bias of the Hg distributions.

In addition, a prominent similarity can be observed between the Hg distributions of Fig. 4a–c. The association of Hgs H, H1, HV, J1c, K1a, T2b completed by Hgs U5a and U5b is totally uniform in all Fig. 4a–c, indicating continuity of a determinant hunter–gatherer–Neolithic farmer component in the Carpathian Basin since the Copper Age. From the early Middle Ages, this stable substrate is complemented by Hgs H3, H1c, and H13a (Fig. 4b), referring to a Caucasian–Near Eastern contribution in CVs 13 and 27 (see Fig. 3b, c). In the ninth and eleventh century Hungarian commoners, this basic layer was enriched by further Hgs of Caucasian–Near Eastern origin H5 and T1a, (Fig. 4b) in the in the ninth century (see also Fig. 3b, c). Figure 4c shows that the resulting ninth to eleventh century complex Hg set also dominates the modern Hungarian population. Besides, we should point at the convincing overlap between Hg distributions of the three Hungarian populations with those of CVs 25 and 34, the former containing Hungarian Neolithic–Bronze Age population in its cluster (see Fig. 3b and OR Clusters-35.dat).

**Fig. 5.**
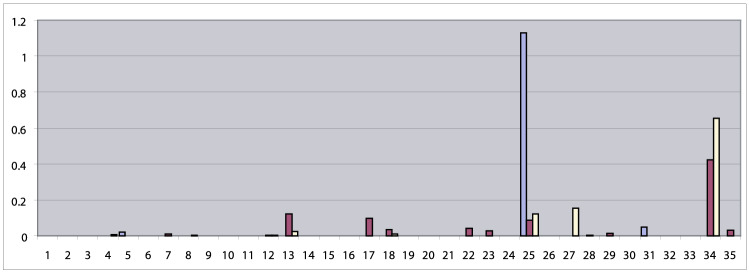
35-dimensional weight vectors of populations in Hungary Hu_CA (blue), ConqC (brown) and modern Hu (yellow). Horizontal axis: serial numbers of CVs. Vertical axis: weights of CVs (colour figure online)

The 35-dimensional weight vectors (coordinates $a_{1} ...a_{N}$ in Eq. 1), calculated for the three Hg distributions above and summarized in Fig. 5, provide more insight into the early admixture processes in Hungary. It is worth mentioning here that the weights providing approximations of biased Hg distributions with minimal error power are not necessarily normalized to one and also can exceed one, as discussed in Supplementary text1.

The most conspicuous phenomena in Fig. 5 are the decreasing weights of CV25 and increasing weights of CV34 with time. We have shown in Chapter 4.3.1 that CVs 25 and 34 have highly similar Hg distributions with shifted frequencies, which can be regarded as Neolithic–Chalcolithic and later Bronze Age versions of European populations with a determinant genetic layer composed of an admixture of Mesolithic hunter–gatherers and Neolithic farmers. Indeed, the gradual decrease of the weights of CV25 and the simultaneous increase of those of CV 34 clearly show the transition between these versions in the substrate population of the Carpathian Basin. The high weight of CV34 in modern Hungarian population shows that this transition is the most important process that determines the genetic structure of recent Hungarians.

A further, Caucasian–Near Eastern–Inner Asian contribution of early Medieval migrations are indicated by the significant weights of CVs 13 and 27, also showing the strong impact of these migrations on modern Hungarians.

Very faint signs of East Eurasian impact from the conquering Hungarians are discernible in the ConqC population; CV35 (Inner-East Asia Late Neolithic Bronze Age), CV29 (Altai–Central Asia Iron Age–Medieval), CV23 (Sintashta Tagar), CV18 (Okunevo), CV7 (Chukchi).

## Discussion

The main goal of our study was to develop a new population genetic approach focusing on jointly propagated Hgs to unravel population histories. Conventional nomenclature of mitochondrial Hgs deduced from sequence data reveal phylogenetic relations, in which closely related Hgs are labeled with similar letter codes reflecting evolutionary geneaology [19]. This consequent nomenclature enables an Hg-based population genetic approach, besides the sequence-based methods. Hg-based population comparison is best applied from deeply classified Hg data determined from full mitogenome sequences; therefore, we first assembled a Eurasian mitogenome database from published data.

Next, we transformed this database by focusing on jointly propagating Hgs which link multiple populations with the objective to reveal their past relationships, as these could have derived only from common ancestry or admixture. Genetic drift and bottleneck effects may distort the footprints of such prehistoric processes, but detectable relations in recently available ancient and modern Hg distributions still may be detected by correlation analysis as shown before [20].

Drift becomes even a dominant factor for isolated populations, leading to the elimination of low frequency Hgs and the emergence of population specific sub-branches. However, these populations typically seldom contributed to major migration and admixture events, which our certainly limited approach is able to detect. As a consequence, these populations typically were absent or underrepresented in our reconstructed common Hg basis, or if such populations appeared, they typically formed their own cluster like Chukchis and Koryaks.

As ancient population relations supposedly involved older phylogenetic subclades these should be taken into account with priority. These considerations led us to model the original Hg distribution of populations by correlated major subclades. As this simplification implicates distortion of the original data we optimized this approach to minimize the bias and demonstrated that this transformation preserved population distances with acceptable accuracy. It follows from our correlation-based selection that an Hg distribution calculated over our 74-dimensional basis represents merely a subset of the real population, derived from ancestors who took part in significant migration processes, whereas region specific Hgs may be eliminated. However, this is not an essential problem when studying major genetic contacts and not peculiarities of populations.

Our algorithm detects all Hg correlations, independently of the date of the process standing in the background of the correlation. For instance, we know from ancient DNA papers, that Hgs arising from the migration of Neolithic farmers to Europe later also took part in the migration of Europeans to South Siberia in the Bronze Age. Our analysis did detect these multiple migration and admixture processes despite the fact, that in reality different derivatives of these Hgs took part in the different steps. Unfortunately, at present we do not yet have enough Hg data from ancient populations, for the complete reconstruction of detailed migration and admixture histories of the more terminal sublineages.

Next, we grouped the populations according to similarity by determining the set of local condensation centre of the Hg-distribution vectors using the SOC algorithm. Providing that the Hg-distribution vectors of our populations construct an appropriately clustered structure, the task of drawing a comprehensive picture of the 172 populations can be reached by representing the groups of similar Hg distributions by their common averaged CVs.

We have shown in Figs. 2 and 3 that many of the 35 CVs found by our search algorithm SOC can be arranged into pairs or triads by their particular similarity. This suggests that the number of CVs could be reduced from 35 to 15–20. Indeed, the algorithm indicates similarly good significance for such amount of CVs, and most of these CVs are really the averaged versions of the pairs and triads shown in Figs. 2 and 3. However, we have shown for the pairs of CVs 30–31, 16–33, 25–34, and 35–29 that the fine differences between them may reveal important geographical and historical particularities.

The closest populations to nearly all CVs are ancient populations. This is indicated by weights of CVs approaching 1 in the linear-combination models of the corresponding nearest ancient populations. We have shown that the CVs can be well interpreted in most cases from archaeogenetic point of view as statistically more credible variants of the nearest ancient Hg distributions. Therefore, the study of the huge number of possible genetic contacts among our 172 populations could be reduced to the study of the impacts of the 35 CVs to any of the populations. In other words, the main genetic features of a given population could be modeled by a 35-dimensional vector containing the weights of the 35 hypothetical genetic “archetypes” optimally modeling its Hg distribution. As the CVs are essentially averages of several similar Hg distributions, they may be considered as statistically more relevant representatives of the wholeness of the populations belonging to their clusters. This may be of particular importance in cases of numerous ancient populations where the small set sizes may cause large bias of the Hg frequencies.

The above interpretations of CVs prompted us to model the populations as linear combinations of CVs, a major novelty of our approach. First, we have shown that original Hg distributions can be adequately modeled by linear combinations of CVs, next we applied this modeling to real populations of the Copper Age, ninth to tenth century and modern Hungarian populations. The models indicate that descendants of Mesolithic hunter–gatherers and Neolithic farmers played dominant roles throughout the history of the Carpathian Basin, while immigrations from the eastern steppe region appear to have minor and temporary impacts on the tenth to eleventh century population.

We have shown that our approach can provide useful insights into the main relationships of the fuzzy structure of archaeogenetic data. We have also shown on ancient and modern data from the Carpathian Basin that the linear combination models of CVs are well interpretable. We think, in the future, this method can reveal important new information from more complete databases, including Y-chromosomal Hgs.

## Supplementary Information

Below is the link to the electronic supplementary material.

Supplementary file1 (XLSX 563 KB)

Supplementary file2 (PDF 84 KB)

Supplementary file3 (XLSX 11 KB)

Supplementary file4 (XLSX 57 KB)

Supplementary file5 (XLSX 16 KB)

Supplementary file6 (XLSX 10 KB)

Supplementary file7 (XLSX 92 KB)

Supplementary file8 (XLSX 36 KB)
