# Supplementary material for: A new linear combination method of haplogroup distribution central vectors to model population admixtures
Source: Mol Genet Genomics. 2022 Apr 11;297(3):889–901. doi: 10.1007/s00438-022-01888-0 (PMC9130205; doi:10.1007/s00438-022-01888-0)
Supplement: Supplementary file 2 — Supplementary file2 (PDF 84 KB) [file 438_2022_1888_MOESM2_ESM.pdf]

ESM_2.pdf: Mathematical description of the linear combination method modeling Hg frequency distributions

Given the D dimensional (Hg distribution) vector $\underline{x}$, and N pieces of D dimensional, not orthogonal central vectors (CVs) $\underline{v}_1 \ldots \underline{v}_N$, we want to determine the scalar weights $a_1 \ldots a_N$, producing the best approximation of the linear combination of $\underline{x}$:

$$\underline{x} = a_1 \underline{v}_1 + a_2 \underline{v}_2 + \ldots + a_N \underline{v}_N + \underline{\varepsilon}, \qquad 1.$$

where $\underline{\varepsilon}$ is the D dimensional error vector.

The requirement that the total error of the approximation H has to be minimized is formulated as

$$H = \varepsilon_1^2 + \varepsilon_2^2 + \ldots + \varepsilon_D^2 = \sum_{k=1}^{D} \varepsilon_k^2 = \min, \qquad 2.$$

where $\varepsilon_1 ... \varepsilon_D$ are the coordinates of $\underline{\varepsilon}$ in the D dimensional (Hg) space.

It follows from Eq. 1 that the kth coordinate of $\underline{\varepsilon}$ is:

$$\varepsilon_k = x_k - \sum_{i=1}^{N} a_i v_{i,k} \quad (k = 1 \ldots D), \qquad 3.$$

therefore H can also be formulated as a function of the weights $a_1 \ldots a_n$:

$$H = \sum_{k=1}^{D} (x_k - \sum_{i=1}^{N} a_i v_{i,k})^2 \ . \qquad 4.$$

To accomplish a gradient search for the weights $a_1 \ldots a_N$ minimizing H, we have to determine the partial derivatives of H as a function of $a_1 \ldots a_N$. It follows from Eq. 2. that

$$\frac{\partial \varepsilon_k}{\partial a_m} = -v_{m,k}, \qquad 5.$$

so

$$\frac{\partial H}{\partial a_m} = \sum_{k=1}^{D} 2\varepsilon_k \frac{\partial \varepsilon_k}{\partial a_m} = 2\sum_{k=1}^{D} \varepsilon_k (-v_{m,k}) \qquad 6.$$

The algorithm based on Equations 1-6 calculates the gradient of H in the space of the weights $a_1 \ldots a_N$ and modifies the solution by a small step in the opposite direction of the gradient in the following steps.

1. The weights $a_1 \ldots a_N$ are initialized by positive random values.
2. The error vector components are calculated using Eq. 3.
3. The partial derivatives of H are determined by Eq. 6.
4. The weights $a_1 \ldots a_N$ are modified in opposite direction of the gradient:

$$a'_m = a_m - \lambda \frac{\partial H}{\partial a_m} \qquad m = 1 \ldots N \quad , \qquad 7.$$

where the scalar λ is a small number controlling the step sizes.

5. In order to avoid negative values of $a_1 \ldots a_N$, the weights are multiplied by a small negative constant immediately when they come into the negative domain. When applying this step, the algorithm searches for pseudo-optimal solution with the constraint that all of $a_1 \ldots a_N$ should be not negative.
6. Steps 1-5 are repeated until the change of *H* reaches a critical minimum value.

There are no restrictions for weights $a_1 \ldots a_N$ when $\underline{x}$ and $\underline{v}_1 \ldots \underline{v}_N$ are arbitrary vectors with real components. However, when $\underline{x}$ and $\underline{v}_1 \ldots \underline{v}_N$ are distribution vectors and $\underline{x}$ is an exact linear combination of $\underline{v}_1 \ldots \underline{v}_N$, i.e.

$$\sum_{k=1}^{k=D} v_{i,k} = 1, \ \sum_{k=1}^{k=D} x_k = 1 \ \text{and} \ \varepsilon_k = 0 \ \ (k=1 \ldots D), \qquad 8.$$

the algorithm approaches the solution fulfilling $\sum_{i=1}^{N} a_i \cong 1$. Nevertheless, this normality cannot be suspected when the linear combination is merely an approximation, i.e. $\varepsilon_k \neq 0$. Obviously, the conditions in Eq. 8 are not fulfilled in our study, therefore the sum of the weights are in the domain of (0.8-1.3) in most of our models, and weights exceeding 1 are also found in certain cases.
